# Real-Time Monitoring Polymerization Reactions Using Dipolar Echoes in ^1^H Time Domain NMR at a Low Magnetic Field

**DOI:** 10.3390/molecules27020566

**Published:** 2022-01-17

**Authors:** Rodrigo Henrique dos Santos Garcia, Jefferson Gonçalves Filgueiras, Luiz Alberto Colnago, Eduardo Ribeiro de Azevedo

**Affiliations:** 1 Instituto de Química de São Carlos, Universidade de São Paulo, CP 369, São Carlos 13660-970, SP, Brazil; rodrigogarciaquimico@yahoo.com.br; 2 Instituto de Química, Universidade Federal Fluminense, Outeiro de São João Batista, Niterói 24020-007, RJ, Brazil; jgfilgueiras@yahoo.com; 3 Instituto de Física, Universidade Federal do Rio de Janeiro, CP68528, Rio de Janeiro 21941-972, RJ, Brazil; 4 Embrapa Instrumentação, Rua XV de Novembro, 1452, São Carlos 13560-970, SP, Brazil; luiz.colnago@embrapa.br; 5 Instituto de Física de São Carlos, Universidade de São Paulo, CP 369, São Carlos 13660-970, SP, Brazil

**Keywords:** time-domain NMR, dipolar echoes, polymerization reaction, epoxy resin, autocatalytic reaction

## Abstract

^1^H time domain nuclear magnetic resonance (^1^H TD-NMR) at a low magnetic field becomes a powerful technique for the structure and dynamics characterization of soft organic materials. This relies mostly on the method sensitivity to the ^1^H-^1^H magnetic dipolar couplings, which depend on the molecular orientation with respect to the applied magnetic field. On the other hand, the good sensitivity of the ^1^H detection makes it possible to monitor real time processes that modify the dipolar coupling as a result of changes in the molecular mobility. In this regard, the so-called dipolar echoes technique can increase the sensitivity and accuracy of the real-time monitoring. In this article we evaluate the performance of commonly used ^1^H TD-NMR dipolar echo methods for probing polymerization reactions. As a proof of principle, we monitor the cure of a commercial epoxy resin, using techniques such as mixed-Magic Sandwich Echo (MSE), Rhim Kessemeier—Radiofrequency Optimized Solid Echo (RK-ROSE) and Dipolar Filtered Magic Sandwich Echo (DF-MSE). Applying a reaction kinetic model that supposes simultaneous autocatalytic and noncatalytic reaction pathways, we show the analysis to obtain the rate and activation energy for the epoxy curing reaction using the NMR data. The results obtained using the different NMR methods are in good agreement among them and also results reported in the literature for similar samples. This demonstrates that any of these dipolar echo pulse sequences can be efficiently used for monitoring and characterizing this type of reaction. Nonetheless, the DF-MSE method showed intrinsic advantages, such as easier data handling and processing, and seems to be the method of choice for monitoring this type of reaction. In general, the procedure is suitable for characterizing reactions involving the formation of solid products from liquid reagents, with some adaptations concerning the reaction model.

## 1. Introduction

Industries increasingly benefit from the use of process analytical technology (PAT) for production control and quality assurance. The usual goal is a comprehensive understanding and thus better control of manufacturing processes. Thus, predictability and reliability of quality should be incorporated into the process; consequently, in the ideal scenario it should be monitored online or in-situ [1,2].

In online or in-situ monitoring it is often necessary to track structural and dynamical changes in real-time. PAT in polymers can be achieved by several physical techniques, among them high- and low-resolution NMR, infrared spectroscopy (FTIR) and Differential Scanning Calorimetry (DSC) techniques [3,4].

Among the NMR methods, ^1^H TD-NMR at a low magnetic field is particularly useful for studying molecular processes where chemical specificity is not necessary. Indeed, because of its high sensitivity to molecular mobility, TD-NMR can detect dynamical chances associated to thermal transitions [5,6,7,8]. In addition, the technique allows to determine other important information such as the ratio between rigid and mobile segments, sizes of molecular clusters in emulsions [9], determination of the degree of crystallinity and crystallite sizes in semicrystalline systems [10], gelation rates in polymer gels [11] and crosslinking and entanglement in elastomers [12,13,14], among others [15,16,17].

^1^H TD-NMR at a low magnetic field is extensively used to determine solid fractions in food. The Solid Fat Content (SFC) [9] method and its fusion curve are used as one of the most important quality parameters for fats. The measured values are used for controlling hydrogenation, interesterification (enzymatic by the enzyme lyase or chemically catalyzed by alkaline metal) and blending, as well as the level of solid fat necessary for the development of new products or replacement of raw material.

^1^H TD-NMR has also been used to monitor polymer crystallization in real-time via T2 measurements. For instance, it was first demonstrated by C. Hertlein et al. [18], who studied the crystallization kinetics of polymer S-Poly(propylene) (sPP), Polyethylene-co-(PEcO) and Poly(ε-caprolactone) (PεCL). In these cases, the reduction of the T2 values, which occurs due the decrease of the polymer chain mobility upon crystallization, is monitored to obtain the crystallization kinetics parameters. The values obtained by ^1^H TD-NMR usually show good agreement with dilatometry and X-ray scattering. 

The advancement of ^1^H TD-NMR methods made possible many other applications. Litvinov and co-workers [19] used the FID and Solid Echo pulse sequences to determine phase composition (mobile fraction, semi-rigid or rigid) and molecular mobility of Nylon 6 fibers. The results satisfactory agreed with results obtained with X-ray diffraction and ^13^C NMR- spectroscopy. Maus [20] used mixed-MSE-CPMG (a combination of the mixed-MSE and Carr–Purcell–Meiboom–Gill pulse sequences) to investigate the solid/liquid ratio and crystallinity of (syndiotactic poly(propylene) (sPP). Maus also investigated the online monitoring of polymer crystallization kinetics and obtained results in good agreement with dilatometric measurements reported for the same sample.

Another example of the real-time monitoring of polymer crystallization was provided by Faria and co-workers [21], who used a dipolar filtered MSE (DF-MSE) pulse sequence [8,22] to monitor one-dimensional crystallization in poly(3-(2′-ethylhexyl)thiophene) (P3EHT) films. In this case, the crystallization does not start from the melt, but from a solid amorphous state, so only little changes in the shape of the ^1^H TDNMR signal are observed. Using a dipolar filter pulse sequence in conjunction with MSE (DF-MSE), these subtle changes could be monitored in order to observe the crystallite growth which was explained using a modified Avrami model for one-dimensional crystallization. 

Oztop [23] determined the crystalline/amorphous fraction obtained with the NMR signal acquired with the MSE pulse sequences of various sugars (glucose, sucrose, etc.) to develop a basic work for a reliable quality-control method. It was demonstrated that the crystallinity of powdered sugars could be predicted using TD-NMR and confirmed the linear dependence of crystallinity with the second moment of the NMR line shape.

There are also many examples showing the real time monitoring of processes using time-domain ^1^H double quantum NMR in a low magnetic field [11,18,24]. As ^1^H double quantum coherences are only created in the presence of ^1^H-^1^H dipolar coupling, the double quantum evolution acts as a filter, keeping only the signal from segments presenting mobility restrictions (rigidified). Hence, the growth of rigidified structures can be monitored in real time. For instance, Saalwächter and co-workers studied gelation in flexible polymer systems [11] and the real time observation of polymer network formation [24]. Another example is the work by Valentin et. al., who successfully used the double quantum filter pulse sequence to selectively detect rigid components and provides a direct probing of the product formation during the cure of an epoxy resin [25].

**Figure 1:**
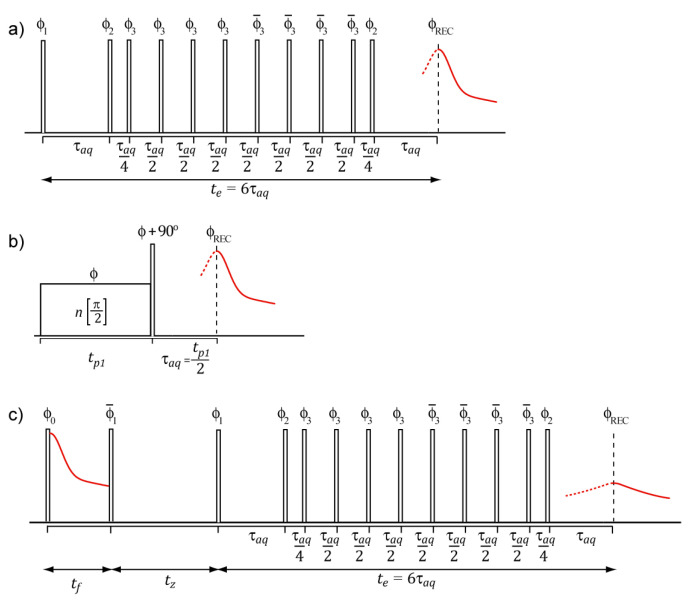
Schemes of the NMR pulse sequences used in this article. (**a**) Mixed-Magic Sandwich Echo (mixed-MSE). (**b**) Rhim–Kessemeier (RK). (**c**) Dipolar Filtered Magic Sandwich Echo (DF-MSE). The phase cycling used in the pulse sequence are provided in references [2,3,4,7]. REC: refer to the receiver phase.

In this article we used ^1^H TD-NMR dipolar echo pulse sequences, shown in Figure 1, for real time monitoring of reactions associated to the cure of a commercial epoxy resin. The cure of a thermorigid resin is a complex process that can be defined as the change in the chemical and physical properties of a given resin/hardener formulation. Because of the formation of a product with rigid molecular segments, understanding the mechanisms and kinetics of cure reactions is essential for a better knowledge of structure–property relationships. The article is organized as follows. First, we discuss the main features related to the dipolar echo pulse sequences mixed-MSE and RK-ROSE. We follow with a discussion about the changes in the signal profile during the polymerization reaction and present the basic data processing needed to extract the parameter used to characterize the rection kinetic. We also discuss a specific reaction model used for characterizing the epoxy cure and establish the meaning of the NMR data in terms of the kinetic parameters of this model. Then, we use the model to fit NMR data acquired with mixed-MSE [26] and RK-ROSE [27] and demonstrate that these experiments are able to bring reliable estimations of the kinetic rate and activation energy of the reactions. Last, we propose the use of the DF-MSE pulse sequence as a simple method for characterizing the reaction, showing its main features and advantages. 

## 2. Results

### 2.1. Signal Profiles during a Polymerization Reaction

The idea behind using ^1^H TD-NMR to probe chemical reactions with the formation of solid products is to follow the evolution of signals from rigid and mobile segments during the reaction. However, ^1^H NMR signals arising from rigid molecular segments have short decay times (≲50 μs) due to the strong ^1^H-^1^H dipolar coupling. Thus, to detect full NMR signals from these segments, it is necessary to start the signal acquisition right after the excitation pulse. This is barely achieved in most of the commercial probeheads, which have typical dead times in the range of 5–30 μs. Thus, a simple π/2—acquisition scheme (denoted here as FID acquisition) frequently implies in the loss of a considerable part of the signal from solid components. One strategy to avoid this dead time issue is to use probeheads with the lowest possible quality factor, but this usually implies in lower sensitivity and requires higher rf power for excitation. An alternative is to use a dipolar echo pulse sequence, capable of refocusing the ^1^H-^1^H dipolar coupling, to produce an echo right out of the dead time region where the solid component is recovered [28]. However, the recovery efficiency of the solid components signals is not 100%, depending on echo times, interpulse delays, pulses errors, etc. Moreover, the shape of the signal can also be affected by the use of a specific pulse sequence or by the experimental set-up [20]. Therefore, different methods can be chosen to obtain better echo recovery efficient or shape accuracy, depending on desired application. 

Here we rely on two basic pulse sequences to refocus the signal from rigid components. The first one is an approach based on the experiments developed by Rhim and Kassemeier in the early 1970s. It consists of applying a continuous wave pulse of duration $t_{p}$ followed by a delay τ and a hard π/2 pulse 90° phase shifted with respect to the CW pulse [28]. Recently, the Rhim and Kassemeier pulse sequence was used without interpulse delays and had a power setup chosen to maximize the signal recovery of solid components at expense of some signal distortion due to the magnitude mode acquisition. This dipolar echo pulse sequence refocuses the dipolar coupling at $\tau_{acq}=t_{p}/2$ after the second pulse and was referred to as Rhim and Kessemeier Radiofrequency Optimized Solid-Echo (RK-ROSE) [27]. The second pulse sequence is the traditional mixed Magic Sandwich Echo (mixed-MSE—Figure 1a) method [26,29]. In this method, a sequence of properly phased pulses refocuses the ^1^H-^1^H dipolar couplings at the same time as eliminating the interference of linear spin interactions such as magnetic field inhomogeneities, chemical shielding, heteronuclear dipolar interactions and local susceptibility variations. Because it can be acquired in the phase mode, the shape analysis of the mixed-MSE echo provide a more reliable way to estimate dipolar coupling second moment, which is particularly important for applications relies on the analysis of the signal shape [30,31]. However, because of the large number of pulses and interpulse delays, the minimum echo time is limited in mixed-MSE, which may compromise the recovery efficiency of signals arising from rigid segments.

**Figure 2:**
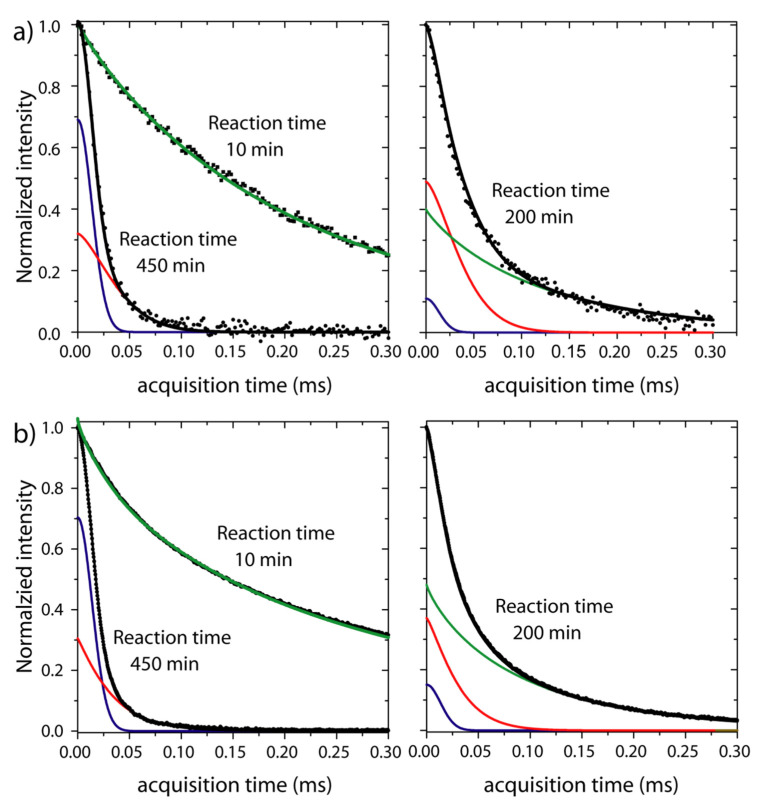
Example of deconvolution of dipolar echoes signals obtained with mixed-MSE (**a**) and RK-ROSE (**b**) pulse sequences using Equation (1). The signals shown in (**a**,**b**) were acquired at the indicated reaction times during the cure of the epoxy resin at 293 K. Note that the differences in the signal to noise between the mixed-MSE and RK-ROSE are due to the differences in the number of scans and echo times as shown in the methods section. Both pulse sequences have similar efficiencies at this set-up condition.

As already mentioned, both RK-ROSE and mixed-MSE pulse sequences allow for differentiation between signals from rigid and mobile segments. Thus, both methods can be used for probing the emergence of rigid segments and/or the disappearing of mobile ones. This is the case of the curing reaction of the epoxy resin, where the formation of the solid products occurs at the expense of the liquid reagents. During such processes, an epoxy resin is converted into crosslinked thermosetting networks, and the thermosetting polymer properties depend on the extent of the chemical reactions that occur during cure and the resin morphology [32]. Briefly, epoxy resin cure goes from a liquid state to a gel point, then turns to rubber, and finally reaches the vitrification point, where it is converted to glass [32,33]. The effect of these processes on the RK-ROSE and mixed-MSE dipolar echoes is shown in Figure 2, where normalized half-echoes signals acquired at several reaction times are presented. The RK-ROSE and mixed-MSE correspond to two different reactions, but with the same relative amount of resin and hardener and at the same temperature. The general behavior of the signals is similar for both type of experiments. The first signal is acquired only after the homogenization of the reagents (~2 min) and temperature equilibration (~10 min). At shorter reaction times (10 min), the signal is comprised of a slow decaying signal associated to ^1^H nuclei in the mobile segments of the reagents. As the reaction takes place (reaction time of 200 min, Figure 2b), the slow decaying fraction of the signal decreases while the fast-decaying fraction increase. When the reaction finishes (reaction time of 450 min in Figure 2a), a fast-decaying signal is observed, as is typical for rigid segments. 

To provide a more quantitative analysis, we refer to the work by Mauss et. al. [20] who decomposed the mixed-MSE signal from a semicrystalline polymer above its glass transition temperature $T_{g}$ in three components, associated to the crystalline (rigid), amorphous (highly mobile) and interfacial (with intermediate mobility) regions. The signals from rigid molecular segments can be well described by the so-called Abragam function, i.e., a multiplication between a gaussian and a sinc function (Van Vleck theory [34]). The signals from intermediate and highly mobile segments are described by modified exponential functions (stretched or compressed), but with different time constant and shape parameters [20]. In summary, the NMR signal can be represented by the following function:

(1) $$\frac{s\left(t\right)}{s\left(0\right)}=f_{r}e^{-{\left(\frac{t}{T_{2r}}\right)}^{2}}\frac{sin\left(bt\right)}{bt}+f_{i}e^{-{\left(\frac{t}{T_{2i}}\right)}^{\nu_{i}}}+f_{m}e^{-{\left(\frac{t}{T_{2m}}\right)}^{\nu_{m}}}$$

where $f_{r}$, $f_{i}$ and $f_{m}$ are the fractions associated to rigid, intermediate and mobile components, respectively. $T_{2r}$, $T_{2i}$, $T_{2m}$, $\nu_{i}$ and $\nu_{m}$ are the respective shape parameters. It is worth to stress that in a low field NMR the signal has significant contribution from static field inhomogeneities. Thus, despite the signal fitting being performed within the 0−300 μs acquisition time window, the shape parameters of mobile components may still have a minor contribution from static field inhomogeneities. 

Here we will use the same model to describe the ^1^H NMR signal, but with the signal from the rigid segments assigned to the solid polymer formed during the cure and the mobile component accounting for the liquid reagents. The segments with intermediate mobility are attributed to polymer chains with restricted mobility in the solid product, for instance, chains in the solid to liquid interface, chain segments with local mobility such side groups or remaining small segments such as oligomers. 

Figure 2 (right) illustrates the fit of normalized signals acquired at 293 K with RK-ROSE and mixed-MSE 10 min and 450 min after the polymerization started. For RK-ROSE the absolute value of the signals is shown (magnitude mode acquisition), while for mixed-MSE the real part of phase adjusted signals are exhibited (phase mode acquisition). After 10 min of reaction, the signal is well adjusted using only the last term of Equation (1) (green curve, $T_{2m}=\left(0.24\pm 0.01\;\right)\;\mathrm{ms}$, $\nu_{m}=0.72\pm 0.05,$ $f_{m}=1.00\pm 0.02$ for RK-ROSE and $T_{2m}=\left(0.21\pm 0.01\;\right)\;\mathrm{ms}$, $\nu_{m}=0.92\pm 0.02,$ $f_{m}=0.99\pm 0.03$ for mixed-MSE), showing that there is no significant rigid phase at this reaction time and temperature, i.e., the signal is only associated to the reagent. On the other hand, after 450 min of reaction only rigid components are observed in the signal. As shown in Figure 2, this fast component can be adjusted using the first and the second terms of Equation (1), i.e., a gaussian-type decay (blue curve, $T_{2r}=\left(0.0135\pm 0.0002\right)\;\mathrm{ms}$, $f_{r}=0.70\pm 0.02$ for RK-ROSE and $T_{2r}=\left(0.0135\pm 0.0002\right)\;\mathrm{ms}$, $f_{r}=0.69\pm 0.02$ for mixed-MSE) and a compressed exponential (red curve, $T_{2i}=\left(0.035\pm 0.002\right)\;\mathrm{ms}$, $\nu_{i}=1.20\pm 0.05$, $f_{i}=0.30\pm 0.02$ for RK-ROSE and $T_{2i}=\left(0.045\pm 0.001\right)\;\mathrm{ms}$, $\nu_{i}=1.50\pm 0.05$, $f_{i}=0.31\pm 0.02$ for mixed-MSE). The signal profile does not change for reaction times longer than 450 min (not shown), suggesting that the cure is already completed. As discussed, the existence of an intermediate mobility phase in the final product can be related to restricted local mobility in the solid phase, for instance due to side chain motions or other local segmental reorientations, or remaining oligomers. Thus, the product fraction can be associated to the sum of the rigid and intermediate mobility components, which will be referred simply as solid fraction, i.e., $f_{S}=f_{r}+f_{i}$. Moreover, we should point out that in both experiments, mixed-MSE and RK-ROSE, there is an underestimation of the rigid fraction because the efficient of the pulse sequences in recovering signals from rigid segments is not 100%. This effect can be corrected as suggested elsewhere [31]. Here this is completed by analyzing the dipolar echoes (acquired with mixed-MSE or RK-ROSE) the simple FID signals acquired after the end of the reaction. This FID signal was shifted by the dead time of our spectrometer (12 μs), and then we perform a joint fit of the dipolar echoes and the corresponding FID signals. In this joint fit, the shape parameters in Equation (1) are shared to impose the FID and the dipolar echo with the same shape. Because the amplitudes are free parameters, the fitting provides independent values of $f_{r}$, $f_{i}$ and $f_{m}$ for the dipolar echo and the FID signals. Thus, it is possible to calculate the ratio between the amplitude parameters associated to dipolar echoes and the FID to estimate a correction factor α, which gives how much of the solid signals is lost due to the acquisition with mixed-MSE or RK-ROSE. For the experiments conducted as a function of temperature, this procedure was performed at each temperature. This correction factor was used to correct the values of $f_{S}=f_{r}+f_{i}$ in all data presented here.

Figure 2 (left) illustrates the fit of normalized signals acquired 200 min after mixing the reagents. In this case, the best fit is achieved using all three components of Equation (1) (blue curve, $T_{2r}=\left(0.0135\pm 0.0002\right)\;\mathrm{ms}$, $f_{r}=0.15\pm 0.01$, red curve, $T_{2i}=\left(0.035\pm 0.002\right)\;\mathrm{ms},$ $\nu_{i}=1.2\pm 0.1$, $f_{i}=0.37\pm 0.03$, green curve, $T_{2m}=\left(0.10\pm 0.05\right)\;\mathrm{ms}$, $\nu_{m}=0.84\pm 0.05$, $f_{m}=0.48\pm 0.05$ for RK-ROSE and blue, $T_{2r}=\left(0.0135\pm 0.0002\right)\;\mathrm{ms}$, $f_{r}=0.11\pm 0.02$, dark yellow, $T_{2i}=\left(0.044\pm 0.004\right)\;\mathrm{ms},$ $\nu_{i}=1.5\pm 0.1$, $f_{i}=0.49\pm 0.03$, magenta, $T_{2m}=\left(0.12\pm 0.04\right)\;\mathrm{ms}$, $\nu_{m}=0.90\pm 0.05$, $f_{m}=0.40\pm 0.06$ for mixed-MSE), as shown individually in Figure 2b. 

Changes in the molecular dynamics of the mobile component throughout the reaction can be observed monitoring the $T_{2m}$ values. $T_{2m}$ values decrease for longer reaction times, suggesting an average slowdown of molecular motions in the mobile phase. This is somewhat expected because the formation of rigid components increases the local molecular constraints, reducing the molecular mobility of the remaining mobile segments. Indeed, $T_{2m}$ has been extensively used to probe reaction kinetics using ^1^H TD-NMR [1]. However, kinetic models for describing polymerization reactions mostly rely on monitoring changes in the reagent and product concentrations. Thus, a more straightforward analysis can be completed using $f_{r}$, $f_{i}$ and $f_{m}$.

### 2.2. Autocatalyzed and Non-Catalyzed Reaction: Relationship between NMR Parameters and Reaction Kinetic Parameters

To obtain the kinetic parameters of the polymerization reaction we first need to establish the meaning of the NMR data in terms of the kinetic parameters. As mentioned, in the curing reaction of epoxy resins, the formation of the solid products occurs at the expense of the liquid reagents. Thus, $f_{m}$ and $f_{s}=f_{r}+f_{i}$ can be associated to the relative reagent, $A\propto f_{m}$, and product, $P\propto f_{r}+f_{i}$, concentrations. Therefore, assuming a model for the polymerization reaction, it would be possible to obtain the kinetic parameters by fitting the reaction time dependence of $f_{m}$ or $f_{r}+f_{i}$. 

Epoxy cure is usually described in terms of noncatalyzed or autocatalytic single step reaction models [14]. The analysis of such reactions using DSC makes it possible to distinguish the contribution of noncatalyzed and autocatalyzed paths by performing dynamic or isothermal experiments, respectively [33,35]. Here, the experiment is essentially isothermal, so we would expect an autocatalytic reaction to prevail. Nonetheless, despite the external temperature of the sample being kept constant, a relatively large sample volume (~0.5 cm^3^) is used, making it difficult to completely avoid internal temperature gradients due to the exothermal character of the reaction, so noncatalytic paths cannot be ruled out. Thus, we build upon a model described in reference [36] that assumes autocatalytic and noncatalytic paths occurring simultaneously. In this model, the reagent and product concentrations are given by:

(2) $$A\left(t\right)=\left(A_{0}+q\right)\frac{A_{0}}{A_{0}+qe^{\left[k_{c}\left(A_{0}+q\right)t\right]}};\;P\left(t\right)=1-A\left(t\right)$$

Here t is the reaction time, $A_{0}$ is the initial reagent concentration and $k_{c}$ is the average reaction rate at a given temperature. The parameter q assumes different meaning for autocatalyzed and noncatalyzed paths, being equal to the initial product concentration, $q=P_{0}$ for autocatalyzed and $q=\frac{k_{0}}{k_{c}}$ for noncatalyzed reactions. $k_{0}$ is the initial reaction rate. 

As already discussed, one may establish a direct correlation between the reagent concentration and the mobile component of the signal, $A\propto f_{m}$, as well as between the product concentration and the solid component, $P\propto f_{S}=f_{r}+f_{i}$. Thus, the curves of $f_{m}$ or $f_{S}$ as a function of the reaction time could be fitted to obtain $A_{0}$, $k_{c}$ and q. However, for higher temperatures one might observe a slow decaying component in the signal that is not associated to the reagent, but to mobile segments in the product. Fortunately, this component can be identified as a mobile fraction kept constant after the reaction is finished. Moreover, it can be taken into account by adding a constant term, $f_{\infty }$, in the fitting function. Hence, the reaction time dependence of $f_{m}$ can be fitted by:

(3) $$f_{m}\left(t\right)=\left(f_{0}+q\right)\frac{f_{0}}{f_{0}+qe^{\left[k_{c}\left(f_{0}+q\right)t\right]}}+f_{\infty };\;\;f_{s}\left(t\right)=1-f_{m}\left(t\right)$$

with $k_{c}$ and q assumed as free fitting parameters and $f_{0}$ obtained as the initial and final $f_{\infty }$ mobile fractions. 

### 2.3. Reaction Kinetic Parameters Extracted from Dipolar Echoes 

In order to probe the dependence of the mobile fraction on the reaction time, we acquired a series of dipolar echoes during the epoxy cure. The dipolar echoes were acquired using mixed-MSE and RK-ROSE in different batches, but with the same proportion of resins and hardeners as well as the same temperature. The signal acquisition started 15 min after mixing the reagents to assure temperature equilibration in the sample. The resulting curves of $f_{m}$ versus the reaction time, $f_{m}\left(t\right)$, were fitted using Equation (3) to obtain $k_{c}$ and q. The fitting procedure used to obtain these parameters was: first, we manually deconvolute the mixed-MSE and RK-ROSE signals at the shortest and the longest reaction times to obtain the shape and amplitude parameters $f_{r}$, $f_{i}$, $f_{m}$, $T_{2r}$, $T_{2i}$, $T_{2m}$, $\nu_{i}$ and $\nu_{m}$. Note that this fitting procedure already provides the values for $f_{0}$ and $f_{\infty }$. Then, these values were used as input values for an automated fit procedure using the origin lab software to obtain the parameter values for each reaction time. 

**Figure 3:**
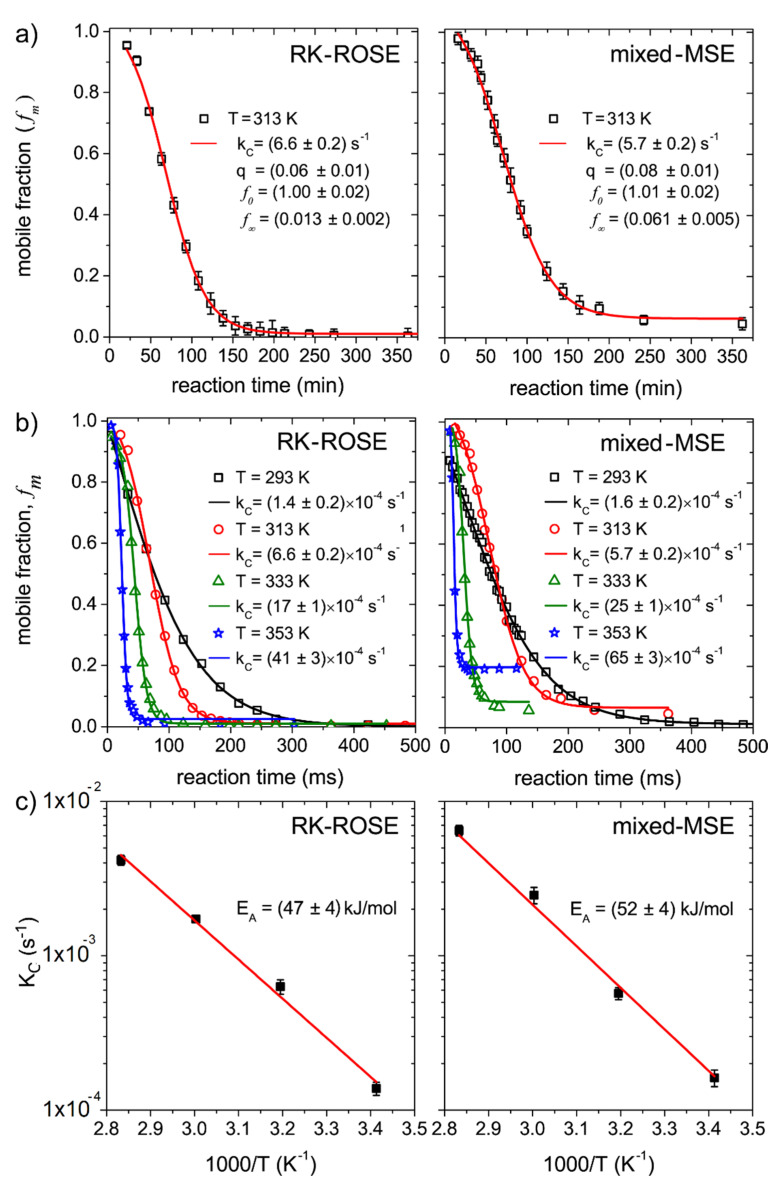
(**a**) Mobile fractions, as function of the reaction time during the epoxy cure, obtained from the deconvolution of signals acquired by the mixed-MSE (right) and RK ROSE (left) pulse sequences at 313 K. (**b**) Same as in (**a**) for 293 K, 313 K, 333 K and 353 K. (**c**) Arrhenius plot of the $k_{c}$ values estimated from the fits shown in (**b**). The error bars were omitted in (**b**), but they are in the same order as those shown in (**a**).The meaning of $k_{c},\;q,\;f_{0},\;f_{\infty }$ are describe in the main text.

Figure 3a shows the plot of the mobile fractions $f_{m}\left(t\right)$ as a function of the reaction time obtained from mixed-MSE and RK-ROSE data at 313 K. Even though the curves obtained by mixed-MSE and RK-ROSE correspond to different batches of samples, the $k_{c}$ values obtained are similar, since the reactions were performed at the same temperatures. In both cases the values obtained for q are close to zero. As mentioned, for autocatalytic reaction the q parameter is equal to the initial product concentration $\left(q=P_{0}\right)$. Because the initial product concentration should be quite small, the lower q obtained suggests the reaction is predominantly of autocatalytic, as expected for an isothermal process. The values of $f_{\infty }$ are different from zero, indicating that part of the segments remain mobile after the end of the reaction. As mentioned, this corresponds to molecular segments in the product, such as pendant groups, chain ends and oligomers, which contributes as intermediate mobility components at the lower temperature (293 K, see Figure 3), but have their mobility increased at 313 K. This remaining mobile component is more evident in the mixed-MSE data due to its ability of refocusing field inhomogeneity effects. We shall consider that even though these segments can contribute to a significant fraction of the signal, they do not hinder the analysis of the reaction kinetics, as they remain constant after the reaction is completed. 

Figure 3b shows the plot of the mobile fractions, $f_{m}\left(t\right),$ as a function of the reaction time, obtained from mixed-MSE and RK-ROSE data at 293 K, 313 K, 333 K and 353 K. The curve fit using Equation (3) provides the $k_{c}$ values at each temperature, as shown in the insets. The temperature dependence of $k_{c}$ is shown in the Arrhenius plot of Figure 3c. Activation energies of $E_{a}=\left(\;47\;\pm \;4\;\right)\;\mathrm{kJ}\;/\;\mathrm{mol}$ and $E_{a}=\left(\;52\;\pm \;4\;\right)\;\mathrm{kJ}\;/\;\mathrm{mol}$ were obtained from the RK-ROSE and mixed-MSE data, respectively. Such values for the activation energies are in good agreement with literature values for similar samples [25,35]. 

### 2.4. Reaction Kinetics Parameters from Dipolar Filtered Magic Sandwich Echo (DF-MSE)

In the last section we showed how dipolar echo pulse sequences can be used to extract kinetic parameters. Nevertheless, performing the signal deconvolution to obtain the mobile, intermediate and solid fractions for each reaction time and temperature can be a quite tedious task and add significant fitting errors to the data. A possible strategy to avoid this is to acquire the data using a pulse sequence that selects only signals from mobile or rigid components or allow to separate these signals in a proper manner. This approach permits an intensity-only analysis, requiring minimal processing effort. For instance, any dipolar filter pulse sequence that suppresses the signal from the rigid phase [37,38,39] can be used to obtain an echo arising only from the mobile components. The amplitude of such an echo, normalized by the corresponding full echo signal, can be plotted as a function of the reaction time to obtain curves similar to those shown in Figure 3b, but without further need of data processing. It is also possible to use a pulse sequence to suppress the mobile phase reaching only the signal from the rigid components, which could also be monitored as a function of the reaction time to probe the polymerization reaction. The most common filter to keep the signal from the rigid phase is the double quantum filter, which only selects signals from dipolar coupled spins, i.e., stiffened segments [40]. Such an approach was already used by Valentin and co-workers to probe epoxy polymerization reactions [25].

Here we discuss an approach based on a simple pulse sequence named Dipolar Filtered Magic Sandwich Echo (DF-MSE); see Figure 1 and reference [8] for details. In its simplest form, DF-MSE is comprised by a Goldman–Shen dipolar filter of duration $t_{f}$ [38,39] followed by the mixed-MSE pulse sequence. At the shorter possible filter time $t_{f0}$ (here 3.5 μs) the Goldman–Shen period has no effect, so a standard mixed-MSE echo is obtained. As already discussed, this signal may contain contributions from both the solid and mobile components with the solid component signal somewhat reduced due to the finite efficiency of the pulse sequence, i.e., $S_{DF-MSE}\left(t_{f0}\sim 0\right)=S_{m}+\alpha S_{s}$, with α≤1 accounting for the reduction in the echo intensity associated to the solid component. Note that α does not change with reaction time. As the filter time is increased the signals from rigid segments are progressively attenuated by the Goldman–Shen dipolar filter, while the signal from the mobile ones is detected without attenuation as long as $T_{2m}\gg t_{f}$. Thus, the detected signal is comprised by the full signal from the mobile components and an attenuated signal from the rigid components. The attenuation can be taken into account considering a factor depending on the Goldman–Shen filter time, i.e., $S_{DF-MSE}\left(t_{f}\right)=S_{m}+\beta_{GS}\left(t_{f}\right)\alpha S_{s}$. If the filter time is long enough $\beta_{GS}\left(t_{f}\right)$ becomes equal to zero, meaning the signals from rigid components are suppressed. This limit is easily identified by the absence of fast decaying signals in the DF-MSE echo. Thus, the ratio between the DF-MSE echo intensities at long and short Goldman-Shen filter times becomes:

(4) $$f_{DF-MSE}\left(t_{f}\right)=\frac{S_{DF-MSE}\left(t_{f}\right)}{S_{DF-MSE}\left(0\right)}=\frac{S_{m}}{S_{m}+\alpha S_{s}}+\frac{\beta_{GS}\left(t_{f}\right)\alpha S_{s}}{S_{m}+\alpha S_{s}}=\gamma f_{m}+f_{GS}\left(t_{f}\right)$$

Therefore, for a filter time adjusted such as $\beta_{GS}\left(t_{f}\right)=0$, the $f_{DF-MSE}$ fraction has the same behavior of $f_{m}$ concerning the dependence with the reaction time. Thus, it can also be fitted using Equation (3) to obtain the reaction kinetic parameters. 

**Figure 4:**
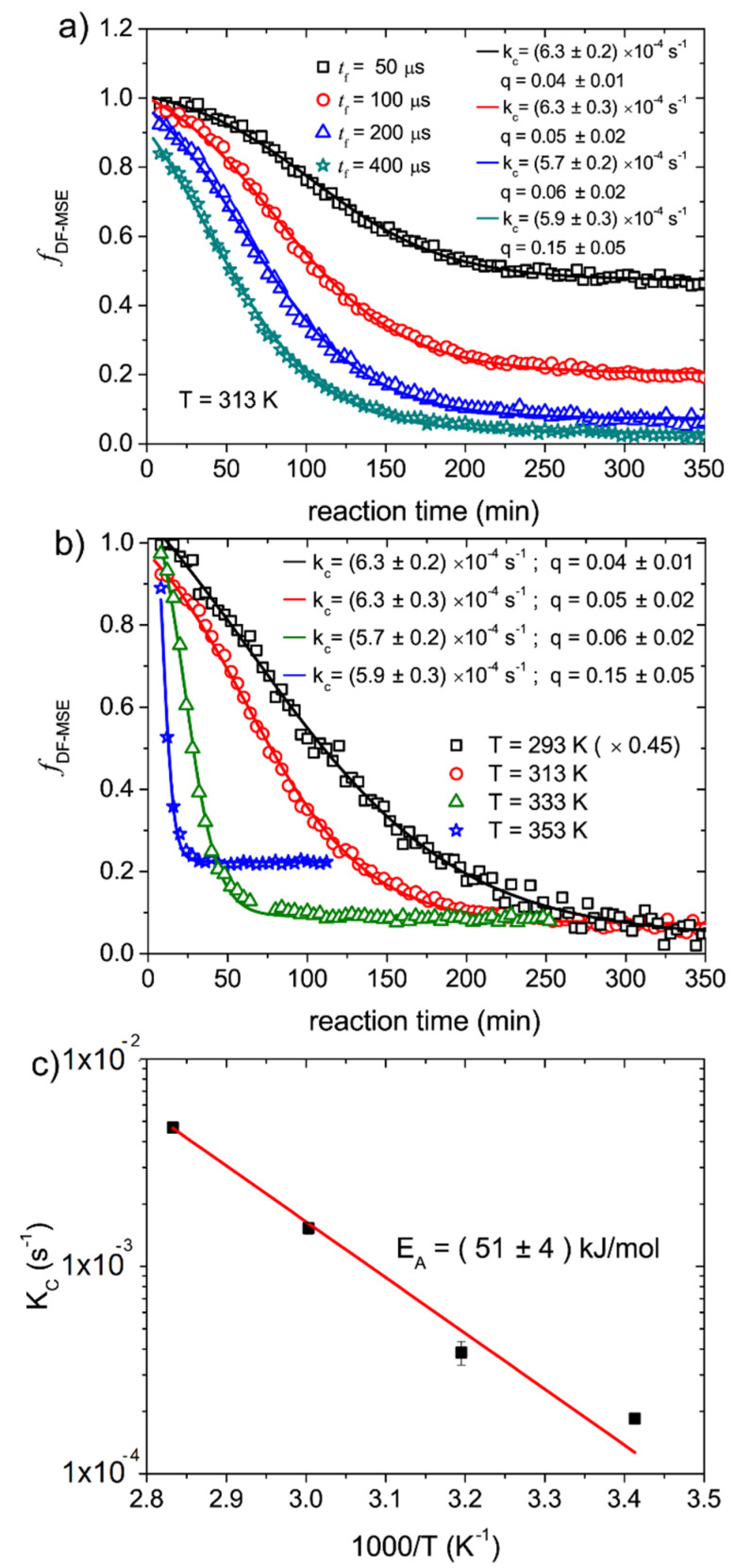
(**a**) Mobile fractions as function of the reaction time during the epoxy cure, calculated from DF-MSE experiments with different filter times. Best fit curves using Equation (3), as well as the obtained values for $k_{C}$ and q, are also shown. The values obtained for $f_{0}$ are all equal to 1.0±0.1. (**b**) Mobile fractions as a function of the reaction time during the epoxy cure at different temperatures calculated from DF-MSE experiments. For better visualization, the curve for 293 K was scaled up by a factor of 0.45. Best fit curves using Equation (3), as well as the obtained values for $k_{C}$ and q, are also shown. The values obtained for $f_{0}$ at all temperatures using the first mixed-MSE echo are equal to 1.01±0.01. The drop in $f_{DF-MSE}$ due to the partial suppression of the mobile phase was taken into account by the γ factor in Equation (3). (**c**) Arrhenius plot of the $k_{c}$ values obtained from the fits shown in (**b**).

Figure 4a show $f_{DF-MSE}$ fractions acquired with filter times of $t_{f}=50\;\mu s,\;100\;\mu s,\;200\;\mu s,\;400\;\mu s$ as a function of the reaction times for the epoxy cure carried out at 313 K. The curves show similar decay shapes, but different plateau values. Another feature observed in Figure 4 is the reduction of the initial $f_{DF-MSE}$ fractions as the filter time increases. This is a result of the relative short $T_{2m}$ values, so at longer filter times part of the mobile component signals is also filtered out by the Goldman–Shen pulse sequence. This attenuation of the mobile signals is taken into account the γ factor in Equation (4). Due to the partial filtering of the solid components at shorter filter times, i.e., $t_{f}=50\;\mu s$ and 100 μs, the plateau is associated to both the $f_{GS}\left(t_{f}\right)$ contribution and the remaining mobile component $f_{\infty }$ as observed in the mixed-MSE experiments. At longer filter times, so $\beta_{GS}\left(t_{f}\right)=0$, the plateau value is only related to $f_{\infty }$, but it can assume a different value because of the partial attenuation of the mobile component. These features can be observed in Figure 4a, where a progressive decrease of the plateau value and initial fractions are observed as the filter time increases. Fitting the curves using Equation (3), we obtained almost the same values for the kinetic rate $k_{c}$, showing that the terms $f_{\infty }$ and γ suffice to take into account the features discussed above. Despite not being necessary, in order to assure a situation with $\beta_{GS}\left(t_{f}\right)=0$ and to minimize the attenuation of mobile component signal, we used $t_{f}=200\;\mu s$ in the DF-MSE experiments to probe the epoxy cure. 

Figure 4b shows the $f_{DF-MSE}$ fractions as a function of reaction time for epoxy polymerization reactions carried out at 293 K, 313 K, 333 K and 353 K. The increase of the plateau values with temperature is associated with the gain of molecular mobility in the molecular segments of the product, as already discussed in the case of the mixed-MSE experiments. The fits using Equation (3) are also shown with the fitting parameters presented as inset. The $k_{c}$ as a function of temperature are shown in the Arrhenius plot of Figure 4c. An activation energy of $E_{a}=\left(\;51\;\pm \;5\;\right)\;\mathrm{kJ}\;/\;\mathrm{mol}$ is obtained. This value is in excellent agreement with those obtained from the mixed-MSE and RK-ROSE data and also with the values found in the literature [25]. 

## 3. Discussions

In summary, our analysis showed that mixed-MSE, RK-ROSE and DF-MSE can be used to describe reactions with formation of solid products. The use of DF-MSE is particularly advantageous, since no extensive processing is required. Indeed, the normalization process can be completed in a fully automated way to display the result while the reaction is in progress, making possible a real-time monitoring of this type of reactions. DF-MSE can be used to monitor reactions when there is a formation of a solid product from liquid reagents. We should also mention that other type of dipolar filters, such as as the Magic and Polarization Echo (MAPE) pulse sequence [37], for mobile fraction determination, or double quantum filter, for rigid fraction determination, can also be used to acquire the data, so the same analysis presented here can be conducted. However, the simplicity of the Goldman–Shen filter used in the current DF-MSE version makes the data acquisition also quite straightforward.

## 4. Materials and Methods

Epoxy resin Araldite^®^ with cure time of 90 min was acquired in the local trade of São Carlos-SP, Brazil. The two components (resin/hardener) were first mixed in the proportion of 10:8 (m/m) for its homogenization (about 120 s), as indicated by the manufacturer and was subsequently placed to the sample in the 10 mm NMR tube.

The spectrometer used was the Bruker Minispec ND mq-20 operating at ^1^H frequency of 20 MHz (0.47 T) with a 10 mm probehead (dead time of 11.6 μs). A π/2 pulse length of 2.4 μs and acquisition time of 5 ms were typically used. The typical decay constant time due to static field inhomogeneity in the spectrometer was about 1.5 ms, so all data processing involving signal deconvolution was restricted to the first 300 μs of the signals. The recycle delays were ~5 s, based on the longitudinal relaxation time (T_1_) determined with the inversion recovery pulse sequence [17]. Variable temperature experiments were carried out using the BVT 3000 temperature controller (Bruker). A previous calibration of the sample temperature was performed by placing a thermocouple immersed in silicon oil in the sample position and relating the thermocouple and the BVT indications. RK-ROSE experiments were performed with a long pulse duration of 24 μs (τ_aq_ in Figure 1 equal to the equipment dead time of 12 μs) and 32 scans. Mixed-MSE experiements were performed with an echo time of 100 μs and 16 scans.

## 5. Conclusions

We presented an extensive analysis about the usage of dipolar echo pulse sequences at a low magnetic field for monitoring polymerization reactions with the formation of solid-products from liquid reagents. As proof of principle, we probed the curing reactions of epoxy resins using three different ^1^H TD-NMR dipolar based methods, i.e., mixed-MSE, RK-ROSE and DF-MSE. 

We showed how mixed-MSE, RK-ROSE data can be processed to bring information about the changes in the product and reagents concentration during the reaction. Using a model assuming autocatalytic and noncatalytic reaction pathways, we establish the relationship between reaction and NMR parameters, so its kinetics could be followed and characterized from NMR data. Using this procedure, we determined an activation energy of about 50 kJ/mol for the cure of a commercial epoxy resin. 

In the last part of the article, we applied the DF-MSE method as a straightforward approach for characterizing these types of reactions. By monitoring a simple normalized intensity as a function of the reaction time, we obtained a curve representing the consumption of the reagents during the process. Using a specific reaction model, this curve was fitted to obtain the reaction rates and to estimate the activation energy by monitoring reactions at different temperatures. In the specific case of the epoxy resin cure, we used the aforementioned model and estimated an activation energy of (51±4) kJ/mol, in agreement with the values obtained using mixed-MSE and RK-ROSE as well as with values reported in the literature.
